# Vorinostat (Suberoylanilide Hydroxamic Acid, SAHA) Coordinately Modulates Cell Cycle Regulatory Genes and Intrinsic and Extrinsic Apoptotic Pathway Genes to Induce Apoptosis in Human Glioblastoma, Medulloblastoma, and Acute Lymphoblastic Leukemia Cells

**DOI:** 10.5812/ijpr-173974

**Published:** 2026-09-20

**Authors:** Fraidoon Kavoosi, Masumeh Sanaei

**Affiliations:** 1 Research Center for Non-Communicable Diseases, Jahrom University of Medical Sciences, Jahrom, Iran Jahrom Iran

**Keywords:** Vorinostat (SAHA), Cell Cycle Arrest, Apoptosis, Gene Expression, Neoplasms

## Abstract

**Background:**

Cell cycle progression is regulated by coordinated interplay among cyclin-dependent kinases (CDKs), cyclins, and cyclin-dependent kinase inhibitors (CKIs). In many human cancers, CKIs are epigenetically silenced through promoter hypermethylation and histone deacetylation, contributing to uncontrolled cell proliferation and impaired apoptosis. Restoration of histone acetylation by histone deacetylase inhibitors (HDACIs) promotes the re-expression of epigenetically silenced tumor suppressor genes, thereby modulating cell cycle control and apoptotic pathways. Building on our previous findings with trichostatin A (TSA), the present study evaluated vorinostat (suberoylanilide hydroxamic acid, SAHA) in three human cancer cell lines.

**Objectives:**

To evaluate the effects of SAHA on the expression of genes involved in cell cycle regulation and in the intrinsic and extrinsic apoptotic pathways, and to assess its ability to induce apoptosis in glioblastoma, medulloblastoma, and acute lymphoblastic leukemia cell lines.

**Methods:**

Half-maximal inhibitory concentration (IC_50_) values for SAHA were determined by treating glioblastoma (SF-763), medulloblastoma (D458-Med), and acute lymphoblastic leukemia (BALL-1) cells with a range of drug concentrations, followed by an MTT assay. Cells treated with the corresponding IC_50_ concentrations were subsequently analyzed for apoptosis by flow cytometry and for the expression of cell cycle regulatory genes and genes involved in the intrinsic and extrinsic apoptotic pathways by quantitative real-time polymerase chain reaction (qRT-PCR).

**Results:**

SAHA significantly modulated the expression of most cell cycle regulatory genes and genes involved in the intrinsic and extrinsic apoptotic pathways. These molecular alterations were accompanied by a significant increase in apoptosis across all three cancer cell lines.

**Conclusions:**

SAHA promotes apoptosis by modulating the expression of cell cycle regulatory genes and activating both extrinsic and intrinsic apoptotic pathways, highlighting its potential as an epigenetic therapeutic agent for glioblastoma, medulloblastoma, and acute lymphoblastic leukemia.

## 1. Background

The maintenance of genomic integrity in mammalian cells depends on the ability to halt cell cycle progression in response to DNA damage. The molecular mechanisms that arrest cell division are termed checkpoints. The cell cycle comprises a series of cellular events that drive a cell to produce two daughter cells. In eukaryotes, the cell cycle consists of the G1 phase (G1), synthesis phase (S), G2 phase (G2), and mitotic phase (M); its progression is principally mediated by cyclin-dependent kinases (CDKs) and their regulatory subunits (cyclins) (1). The activity of CDKs (cyclin–CDK complexes) is tightly regulated by a group of proteins termed cyclin-dependent kinase inhibitors (CKIs). Mitogenic stimulation induces D-type cyclins (D1, D2, and D3), which activate CDKs. Cyclin E is an essential regulator of cell cycle progression through G1 (2). In mammalian cells, the cell cycle is controlled by CDK2, CDK4, CDK6, and associated cyclins (3). CKIs are classified into two groups: the INK4 and Cip/Kip inhibitors. The INK4 family includes p16INK4a (p16), p15INK4b (p15), p18INK4c (p18), and p19INK4d (p19), and the Cip/Kip family comprises p21Cip1/Waf1/Sdi1 (p21), p27Kip1 (p27), and p57Kip2 (p57) (4). Recent studies have indicated that CKIs exhibit tumor suppressor activity and are frequently inactivated in various human cancers through different molecular mechanisms (5). A common epigenetic hallmark of cancer cells is CpG island hypermethylation in the promoter regions of tumor suppressor genes (TSGs), accompanied by histone deacetylation, which contributes to their transcriptional silencing. CKIs have been reported to be inactivated by these two mechanisms, resulting in tumorigenesis (6, 7). DNA methylation is established by DNA methyltransferases (DNMTs), whereas histone deacetylases (HDACs) remove acetyl groups from histones, thereby facilitating chromatin compaction and suppression of gene transcription. Conversely, DNMT inhibitors induce DNA demethylation, whereas HDAC inhibitors promote histone acetylation and transcriptional reactivation of silenced genes. DNA methyltransferase inhibitors (DNMTIs) and histone deacetylase inhibitors (HDACIs) induce DNA demethylation and histone acetylation, respectively, leading to reactivation of silenced TSGs (8). In addition to cell cycle arrest, CKIs may sensitize tumor cells to apoptosis through multiple molecular mechanisms, including modulation of death receptor signaling (9). We previously demonstrated that the antitumor activity of the HDACI valproic acid (VPA) is mediated through the intrinsic apoptotic pathway. VPA downregulated class I histone deacetylases (HDAC1, HDAC2, and HDAC3), Bcl-2, Bcl-xL, and Mcl-1 and upregulated p21, p53, Bax, Bak, and Bim, resulting in apoptosis induction in the hepatocellular carcinoma HepG2 cell line (10). Additionally, we showed that trichostatin A (TSA), a histone deacetylase inhibitor, affects apoptosis via reactivation of negative cell cycle regulatory genes (CKIs). TSA increased p21, p27, and p57 and decreased DNMT and HDAC gene expression in the colon cancer LS 180 cell line (11). Furthermore, HDACIs can engage the extrinsic apoptotic pathway through upregulation of death receptor (DR) expression, upregulation of ligands such as tumor necrosis factor-related apoptosis-inducing ligand or Apo 2 ligand (TRAIL/Apo2 L), and reductions in c-FLIP (12). Therefore, cell cycle arrest and apoptosis are closely interconnected cellular responses that collectively preserve genomic integrity under conditions of stress. In addition to suppressing cyclin–CDK activity and preventing cell cycle progression, tumor suppressors such as RB1 and Cip/Kip family members (p21, p27, and p57) influence cell fate decisions by promoting apoptotic signaling when DNA damage or other cellular insults cannot be adequately repaired. Persistent cell-cycle arrest may therefore facilitate activation of both death receptor-mediated and mitochondrial apoptotic pathways, ensuring the elimination of irreversibly damaged cells. Consequently, coordinated regulation of cell cycle checkpoints and apoptotic signaling represents a fundamental mechanism through which anticancer agents exert their therapeutic effects (13, 14). Two principal signaling pathways regulate apoptosis: the extrinsic (death receptor-mediated) pathway and the intrinsic (mitochondrial) pathway. The extrinsic pathway is initiated by the interaction of death ligands with their cognate receptors, including Fas (CD95) and death receptors such as DR4 and DR5. Upon receptor activation, adaptor proteins such as Fas-associated death domain (FADD) are recruited to form the death-inducing signaling complex (DISC), leading to activation of initiator caspase-8 and subsequently of executioner caspase-3 (15). By contrast, the intrinsic apoptotic pathway is governed by the relative activities of pro-apoptotic proteins (e.g., Bax) and anti-apoptotic proteins (e.g., Bcl-2), which regulate mitochondrial outer membrane permeabilization and subsequent cytochrome c release. This process leads to apoptosome assembly, followed by activation of caspase-9 and subsequently caspase-3. Crosstalk between these pathways provides a coordinated mechanism for efficient elimination of damaged or malignant cells (16). Despite accumulating evidence that HDAC inhibitors regulate cell cycle progression and apoptosis, most previous studies have focused on either cell cycle arrest or a single apoptotic pathway. Whether SAHA coordinately regulates cell cycle arrest together with both extrinsic and intrinsic apoptotic pathways across different tumor types remains insufficiently understood.

## 2. Objectives

Because the effects of HDACIs depend on concentration, exposure time, and tissue or cell line (17-19), this study aimed to determine whether vorinostat (suberoylanilide hydroxamic acid, SAHA) coordinately modulates cell cycle regulatory networks and both death receptor-mediated and mitochondrial apoptotic signaling pathways, thereby promoting apoptosis in the human glioblastoma SF-763, medulloblastoma D458-Med, and acute lymphoblastic leukemia BALL-1 cell lines. To address this objective, the expression of representative genes governing cell cycle progression and extrinsic and intrinsic apoptosis was systematically examined. The analyzed genes are summarized in Table 1.

**Table 1.** Genes Investigated in the Present Study According to Their Functional Involvement in Cell Cycle Regulation and the Intrinsic and Extrinsic Apoptotic Pathways

Pathways	Gene/name
**Cell cycle arrest**	RB1, P21, P27, P57, Cyclin D1, CDK4, CDK6, Cyclin E, CDK2
**Extrinsic apoptotic pathway**	TRAIL, DR4, DR5, FAS, FADD, Caspase-8, Caspase-3
**Intrinsic (mitochondrial) pathway**	BAX, BCL2, Caspase-9, Caspase-3

## 3. Methods

### 3.1. Study Design and Setting

This study used three human cancer cell lines: glioblastoma (SF-763), medulloblastoma (D458-Med), and acute lymphoblastic leukemia (BALL-1). These cell lines were purchased from the same source as previously reported, and the cells were maintained under the same culture conditions as previously described (10, 11). All chemicals, reagents, kits, and drugs used in this study were obtained from the same suppliers as previously reported (15). A stock solution of SAHA was prepared by dissolving the compound in dimethyl sulfoxide (DMSO) and was stored at −20 °C until use. Immediately before each experiment, the stock solution was diluted with the appropriate culture medium to obtain the required working concentrations.

### 3.2. Cell Culture and Cell Viability

To determine cell viability and calculate the half-maximal inhibitory concentration (IC_50_) values, the cell lines (SF-763, D458-Med, and BALL-1) were cultured under the same conditions using the culture medium previously described (11). After overnight incubation, the cells were provided with fresh culture medium supplemented with SAHA at concentrations of 0, 1, 2.5, 5, 7.5, 10, and 20 μM. After 24 h of SAHA treatment, cell viability was assessed by the MTT assay, as previously described (11).

### 3.3. Apoptosis Analysis

To assess apoptosis, the cells (SF-763, D458-Med, and BALL-1) were seeded at a density of 3 × 10^5^ cells per well in 96-well plates and incubated overnight. After 24 h, the culture medium was replaced with fresh medium containing SAHA at the corresponding IC_50_ concentration, as determined by the MTT assay (Table 2), for 24 h. Vehicle control cells received the same concentration of DMSO as that used in the SAHA-treated groups. Flow cytometric analysis was then performed as previously described (12).

**Table 2.** Cytotoxic Activity of SAHA in the Investigated Cancer Cell Lines: IC_50_, Log IC_50_, and R^2^ Values

Cell Line	IC_50_ Value	Log IC_50_	R^2^
**SF-763**	12.28	0.9110 to 1.285	0.9800
**D458-Med**	7.741	0.6135 to 1.171	0.9539
**BALL-1**	7.581	0.5234 to 1.246	0.9283

### 3.4. Quantitative Real-Time Polymerase Chain Reaction

Quantitative real-time polymerase chain reaction (qRT-PCR) was performed in all cell lines to determine the expression levels of genes involved in the three pathways presented in Table 3: cell cycle arrest and the extrinsic and intrinsic apoptotic pathways. SF-763, D458-Med, and BALL-1 cells were seeded at 3 × 10^5^ cells/well and exposed to the respective IC_50_ concentrations of SAHA (Table 2) for 24 h. Vehicle control cells received the same concentration of DMSO as the SAHA-treated groups. Total RNA was subsequently isolated from SF-763, D458-Med, and BALL-1 cells using the RNeasy Mini Kit (Qiagen, Hilden, Germany) in accordance with the manufacturer's instructions. The isolated RNA was then treated with RNase-free DNase (Qiagen, Hilden, Germany) to eliminate residual genomic DNA contamination. qRT-PCR was performed as previously described. The primer sequences (20-36) used in this study are listed in Table 3.

**Table 3.** Primer Sequences, Amplicon Sizes, and References for the Genes Analyzed in the Cell Cycle Regulatory, Extrinsic Apoptotic, and Intrinsic Apoptotic Pathways

Primers	Primer Sequences (5' to 3')	Product Length/bp	Reference
	Cell cycle arrest pathway		
**RB1 / Forward / Reverse**	/ GACCGAGAAGGACCAACTGA / AAGGCTGAGGTTGCTTGTGT	176	20
**P21 / Forward / Reverse**	/ ATGTGTCCTGGTTCCCGTTTC / CATTGTGGGAGGAGCTGTGA	121	21
**P27 / Forward / Reverse**	/ AACGTGCGAGTGTCTAACGG / CCCTCTAGGGGTTTGTGATTCT	209	22
**P57 / Forward / Reverse**	/ CGGCGATCAAGAAGCTGTCC / CGGGGCTCTTTGGGCTCT	193	23
**Cyclin D1 / Forward / Reverse**	/ CCAAATCTCAATGAAGCCAGC / AATTCTTTTGGTTCGGCAGCT	73	24
**CDK4 / Forward / Reverse**	/ AAATTGGTGTCGGTGCCTATGG / CACGAACTGTGCTGATGGGAAG	135	25
**CDK6 / Forward / Reverse**	/ TGGAGACCTTCGAGCACC / CACTCCAGGCTCTGGAACTT	149	26
**Cyclin E / Forward / Reverse**	/ AGTGGCGTTTAAGTCCCCTG / ATACAAGGCCGAAGCAGCAA	203	27
**CDK2 / Forward / Reverse**	/ ATTCTGAGATTGACCAGCTCTTCC / CTTGGCTTGTAATCAGGCATAGAAG	100	25
	Extrinsic apoptotic pathway		
**TRAIL / Forward / Reverse**	/ CCCAATGACGAAGAGAGTATGAACA / AAAATCATCTTTCTAACGAGCTGA	80	28
**DR4 / Forward / Reverse**	/ ACCTTCAAGTTTGTCGTCGTC / AACTCTCCCAAAGGGCTATGT	122	29
**DR5 / Forward / Reverse**	/ GGGAGCCGCTCATGAGGAAGTTGG / GGCAAGTCTCTCTCCCAGCGTCTC	182	30
**FAS / Forward / Reverse**	/ GCCAATTCTGCCATAAGC / TTGTCTGTGTACTCCTTCC	115	31
**FADD / Forward / Reverse**	/ CGAGTCCGAAGATTCCTGA / GACCCTCCGGAGTTTATTCA	150	32
**Caspase-8 / Forward / Reverse**	/ AGAAGAGGGTCATCCTGGGAGA / TCAGGACTTCCTTCAAGGCTGC	142	33
**Caspase-3 / Forward / Reverse**	/ GGAAGCGAATCAATGGACTCTGG / GCATCGACATCTGTACCAGACC	146	33
	Intrinsic apoptotic pathway		
**BAX / Forward / Reverse**	/ GCTTCAGGGTTTCATCCAG / GGCGGCAATCATCCTCTG	169	34
**BCL2 / Forward / Reverse**	/ TACTTAAAAAATACAACATCACAG / GGAACACTTGATTCTGGTG	153	34
**Caspase-9 / Forward / Reverse**	/ GCAGGCTCTGGATCTCGGC / GCTGCTTGCCTGTTAGTTCGC	152	35
**Caspase-3 / Forward / Reverse**	/ GGAAGCGAATCAATGGACTCTGG / GCATCGACATCTGTACCAGACC	146	33
**GAPDH / Forward / Reverse**	/ TGTTGCCATCAATGACCCCTT / CTCCACGACGTACTCAGCG	148	36

### 3.5. Statistical Analysis

Data were analyzed using GraphPad Prism version 8.0 (GraphPad Software, San Diego, CA, USA). All results are presented as the mean ± SD of three independent experiments (n = 3). Statistical differences among groups were evaluated by one-way analysis of variance (ANOVA), and a P value < 0.05 was considered statistically significant. Differences between the SAHA-treated and control groups were evaluated using one-way ANOVA, with statistical significance defined as P < 0.05.

### 3.6. Ethical Considerations

This study was approved by the Ethics Committee of Jahrom University of Medical Sciences (Approval No. IR.JUMS.REC.1405.019).

## 4. Results

### 4.1. Effect of SAHA on Cell Viability

The cytotoxic effect of SAHA was evaluated using the MTT assay. Exposure to SAHA resulted in a significant reduction in the viability of SF-763, D458-Med, and BALL-1 cells compared with that of the control groups (P < 0.05) (Figure 1). Table 2 summarizes the IC_50_ values determined for each cell line.

**Figure 1.**
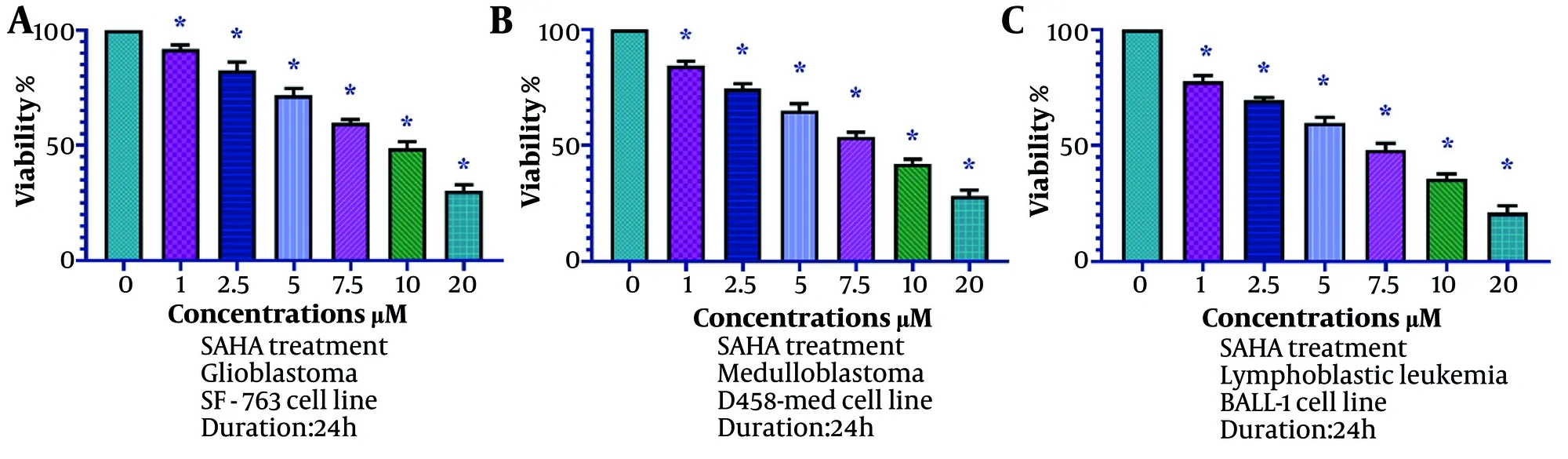
Effect of SAHA on cell viability. SAHA significantly inhibited cell growth. Asterisks indicate significant differences between control and experimental groups (A-C). *P < 0.05 versus control.

### 4.2. Effect of SAHA on Apoptosis Induction

After 24 h of SAHA treatment, apoptosis in SF-763, D458-Med, and BALL-1 cells was quantified by dual staining with Annexin V-FITC and propidium iodide (PI). Flow cytometric analysis demonstrated that SAHA significantly increased apoptosis in all three cell lines (Figure 2) (P < 0.05). The highest and lowest apoptotic rates were observed in D458-Med and BALL-1 cells, respectively (Figure 3).

**Figure 2.**
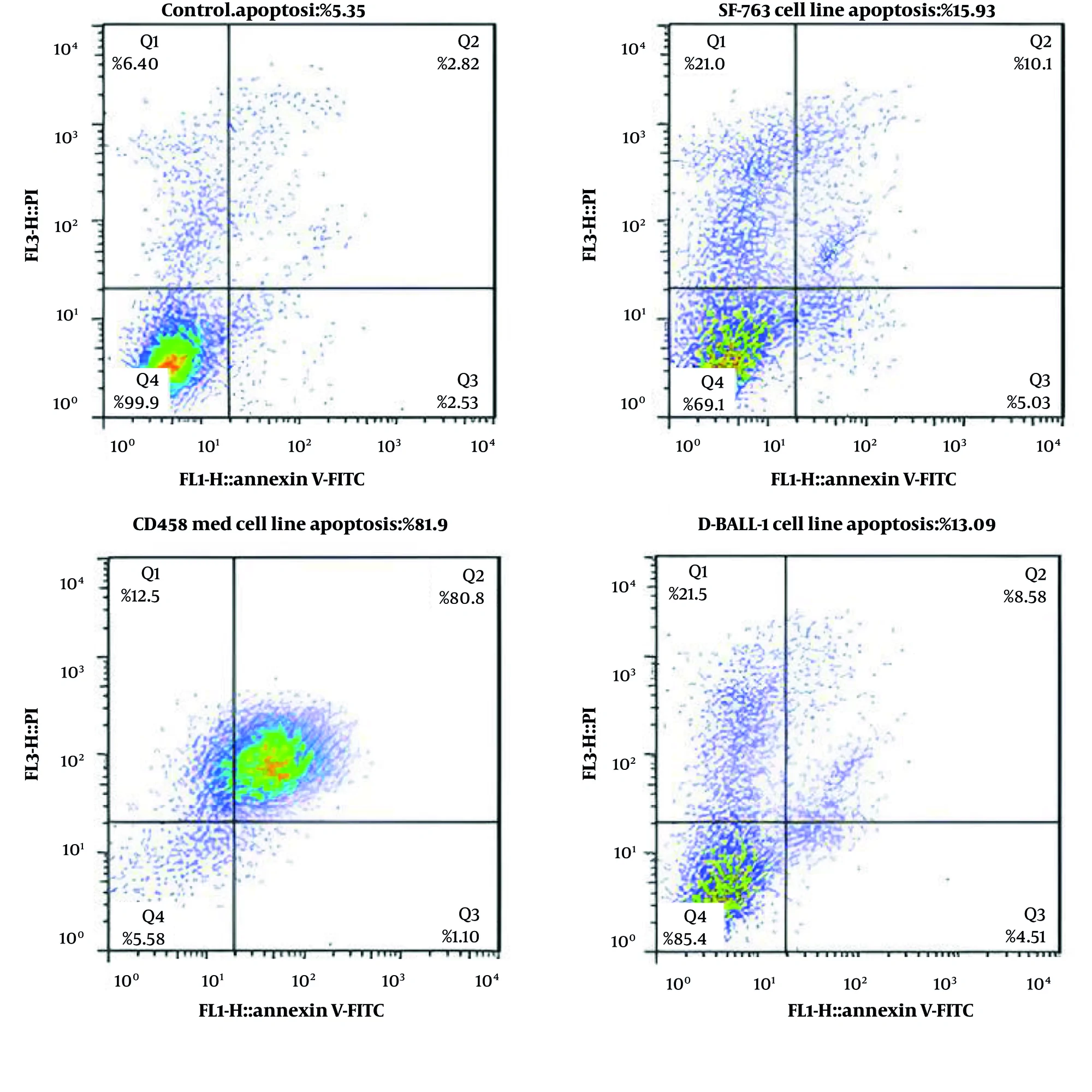
Representative flow cytometry plots showing the effect of SAHA on apoptosis in SF-763, D458-Med, and BALL-1 cells following 24 h of treatment. The percentages shown in the quadrants represent the distribution of viable, early apoptotic, late apoptotic, and necrotic cells in representative samples. Flow cytometry experiments were independently repeated three times (n = 3). Quantitative analysis of apoptosis and statistical comparisons with the corresponding untreated controls are presented in Figure 3.

**Figure 3.**
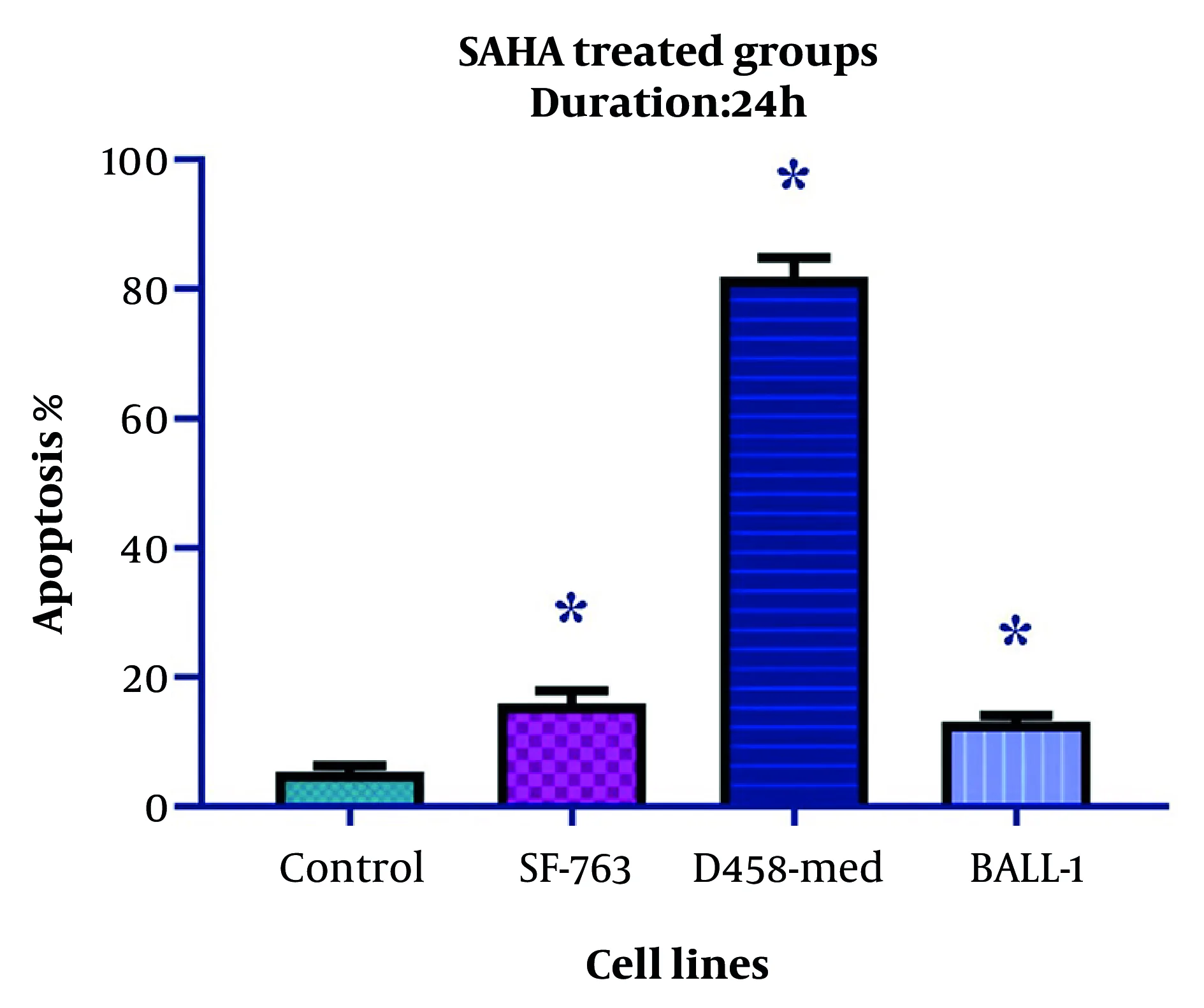
Comparative analysis of SAHA-induced apoptosis among SF-763, D458-Med, and BALL-1 cells following 24 h of SAHA treatment. The highest level of apoptosis was observed in D458-Med cells, whereas the lowest level was detected in BALL-1 cells. Statistical comparisons were performed between each cell line and its corresponding untreated control. The exact P values were SF-763 (***P = 0.0004), D458-Med (****P < 0.0001), and BALL-1 (**P = 0.0031). A P value < 0.05 was considered statistically significant.

### 4.3. Effect of SAHA on Gene Expression

To determine gene expression levels, SF-763, D458-Med, and BALL-1 cells were treated with SAHA, and relative gene expression was evaluated by qRT-PCR. qRT-PCR analysis demonstrated that SAHA induced significant changes in the expression of most genes involved in the three pathways (P < 0.05), except for several genes that did not show statistically significant changes, as presented in Table 4 and Figures 4-6.

**Table 4.** Genes with No Statistically Significant Changes in Expression Following SAHA Treatment in Each Cell Line Across the Investigated Signaling Pathways

Cell Names and Pathways	No Significant Expression
**Medulloblastoma (D458-Med)**	
Cell cycle	P27, P57, CDK4
Extrinsic apoptotic pathway	-
Intrinsic apoptotic pathway	-
**Glioblastoma (SF-763)**	
Cell cycle	P57, CDK6
Extrinsic apoptotic pathway	FADD
Intrinsic apoptotic pathway	Caspase-3
**Lymphoblastic leukemia (BALL-1)**	
Cell cycle	P27, Cyclin D1, CDK6
Extrinsic apoptotic pathway	DR5
Intrinsic apoptotic pathway	Caspase-3

**Figure 4.**
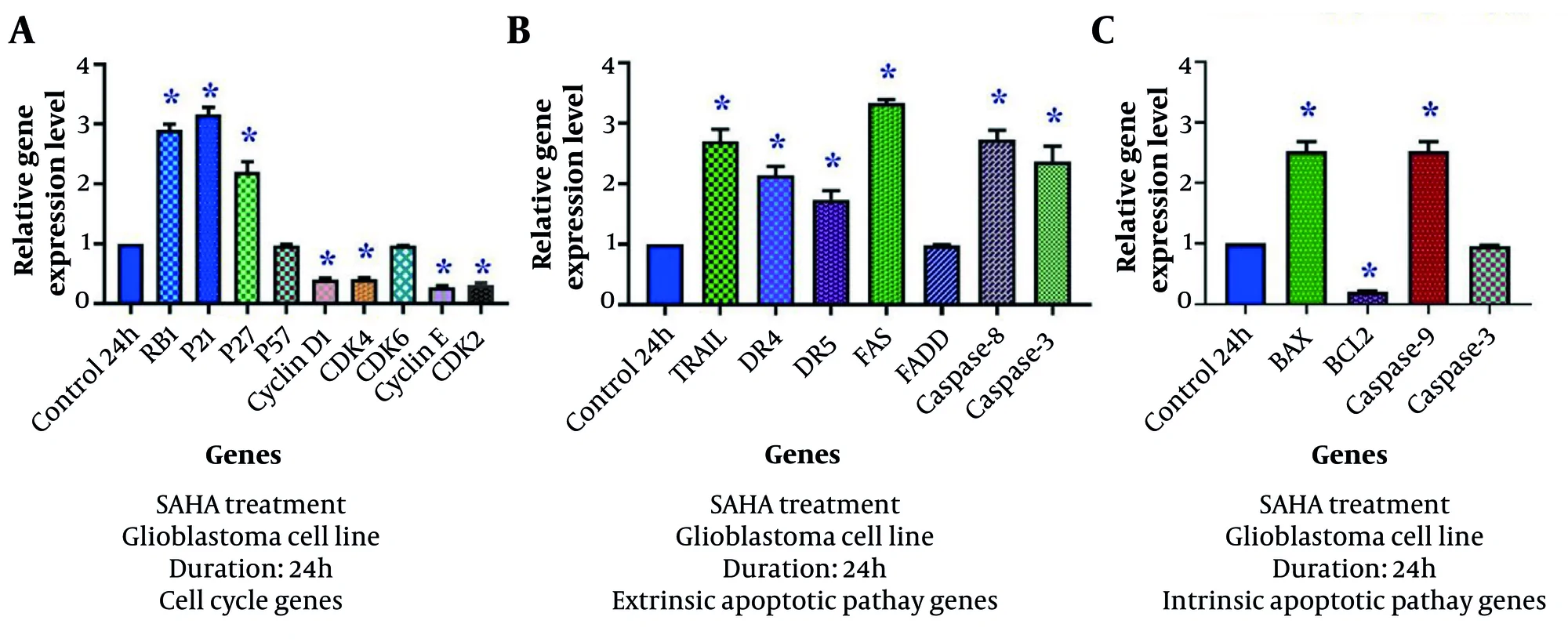
Gene expression changes in the three investigated pathways in the glioblastoma cell line. A, Cell cycle regulatory genes: No significant differences in gene expression were observed for P27 (P = 0.9999) and CDK6 (P = 0.9999), whereas all remaining genes showed highly significant differences compared with the untreated control (****P < 0.0001); B, Extrinsic apoptotic pathway genes: No significant difference in gene expression was observed for FADD (P = 0.9997), whereas all remaining genes showed significant differences compared with the untreated control. DR5 showed ***P = 0.0001, while all other genes showed ****P < 0.0001; C, Intrinsic apoptotic pathway genes: No significant difference in gene expression was observed for Caspase-3 (P = 0.9599), whereas all remaining genes showed highly significant differences compared with the untreated control (****P < 0.0001).

**Figure 5.**
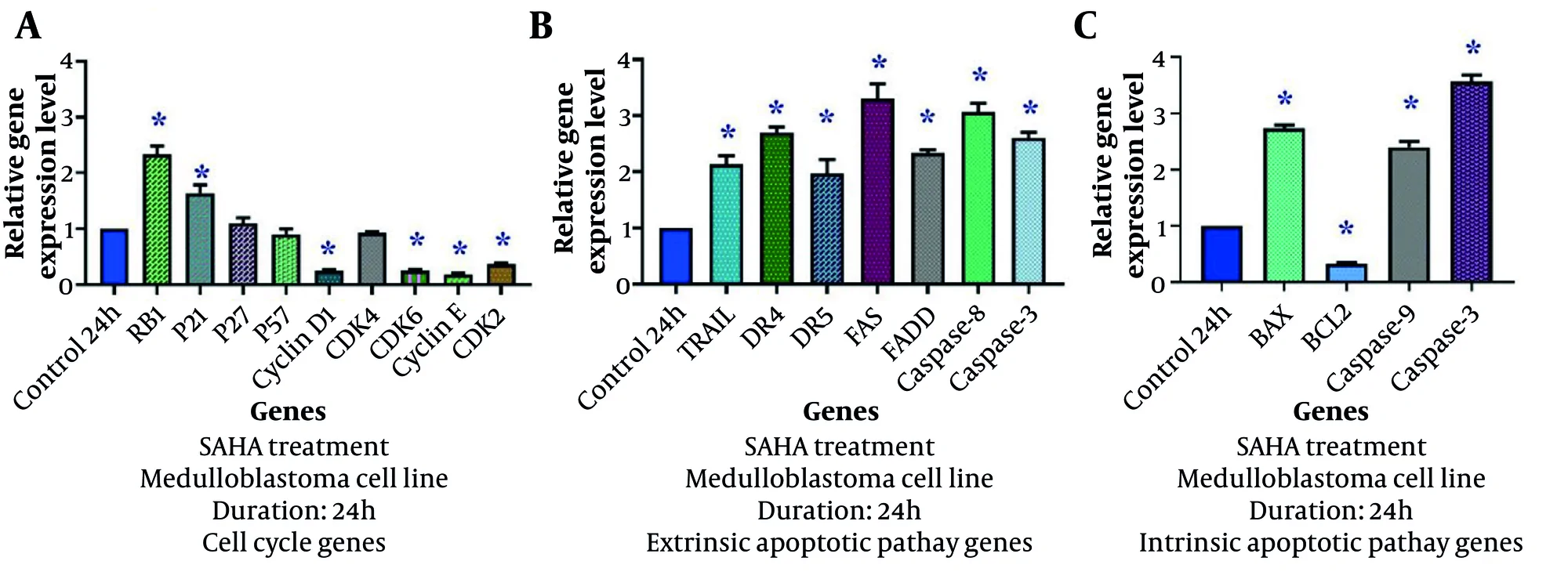
Gene expression changes in the three investigated pathways in the medulloblastoma cell line. A, Cell cycle regulatory genes: No significant differences in gene expression were observed for P27 (P = 0.6043), P57 (P = 0.6533), and CDK4 (P = 0.9069), whereas all remaining genes showed highly significant differences compared with the untreated control (****P < 0.0001); B, Extrinsic apoptotic pathway genes: All analyzed genes showed highly significant differences in gene expression compared with the untreated control (****P < 0.0001); C, Intrinsic apoptotic pathway genes: All analyzed genes showed highly significant differences in gene expression compared with the untreated control (****P < 0.0001).

**Figure 6.**
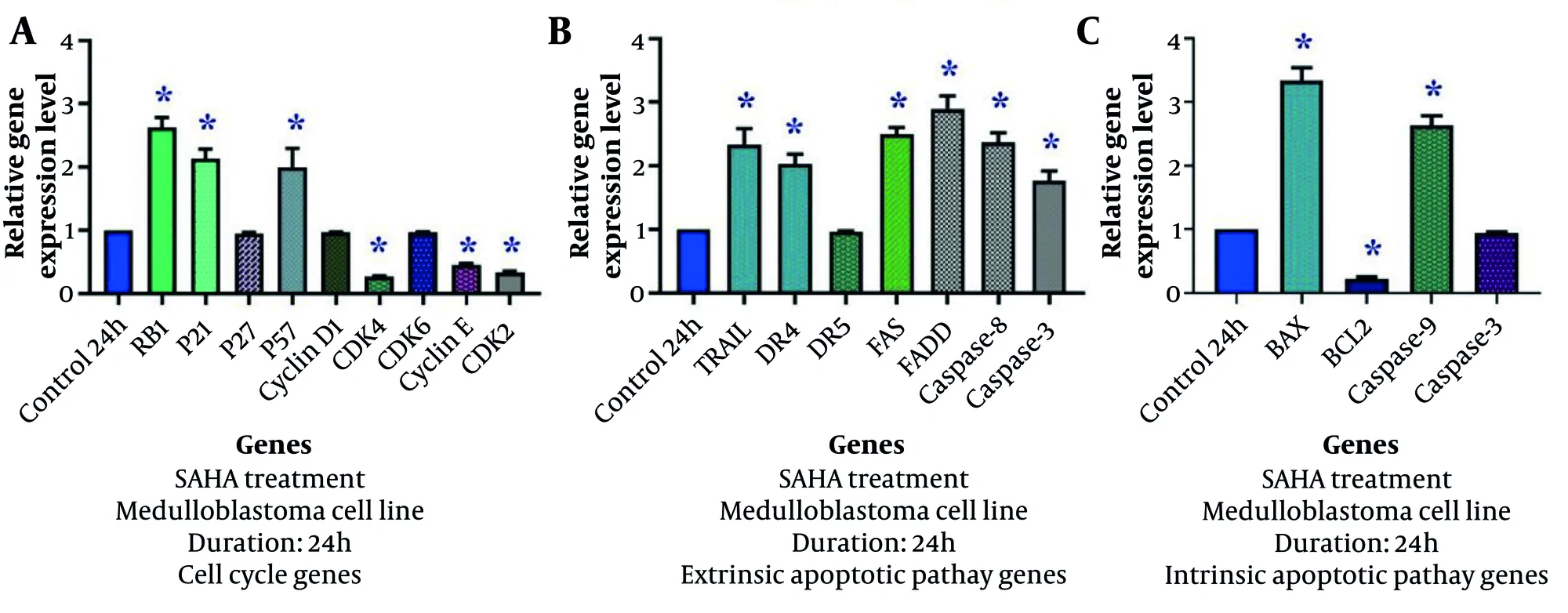
Gene expression changes in the three investigated pathways in the leukemia cell line. A, Cell cycle regulatory genes: No significant differences in gene expression were observed for P27 (P = 0.9970), Cyclin D1 (P = 0.9996), and CDK6 (P = 0.9995), whereas all remaining genes showed significant differences compared with the untreated control. Cyclin E showed ***P = 0.0001, while all other genes showed ****P < 0.0001; B, Extrinsic apoptotic pathway genes: No significant difference in gene expression was observed for DR5 (P = 0.9999), whereas all remaining genes showed highly significant differences compared with the untreated control (****P < 0.0001); C, Intrinsic apoptotic pathway genes: No significant difference in gene expression was observed for Caspase-3 (P = 0.9446), whereas all remaining genes showed highly significant differences compared with the untreated control (****P < 0.0001).

## 5. Discussion

Cancer cells frequently acquire defects in cell cycle control mechanisms involving cyclins, CDKs, and cyclin-dependent kinase inhibitors (CKIs), thereby enabling sustained proliferative activity. Aberrant overexpression of cyclins and CDKs, together with downregulation of CKIs, has been reported in many human malignancies and promotes an uncontrolled G1/S transition (37). Previous studies have demonstrated that HDAC inhibitors, including SAHA and depsipeptide, modulate the expression of multiple genes involved in cell cycle regulation, thereby inducing growth arrest in various cancer cells (38). Consistent with these findings, our previous study demonstrated that the HDAC inhibitor sodium butyrate induced apoptosis through upregulation of CKIs and activation of the intrinsic apoptotic pathway, accompanied by altered expression of BAX, BAK, APAF1, BCL2, and BCL2L1 (Bcl-xL) in hepatocellular carcinoma cell lines (39). As summarized in Table 4 and illustrated by the gene expression profiles in Figures 4-6, the present findings demonstrate that SAHA markedly modulated the expression of genes involved in cell cycle regulation, including upregulation of CKIs and modulation of cyclin/CDK-associated genes, as well as genes associated with the intrinsic and extrinsic apoptotic pathways. In agreement with the present findings, previous studies have demonstrated that SAHA increases p21 (CKI) gene expression in human multiple myeloma cells (40). Similarly, SAHA has been shown to inhibit cell growth and induce cell cycle arrest by modulating cell cycle regulatory proteins, including p21 and p27, in the human breast cancer cell lines MDA-MB-231, MCF7, and MDA-MB-435 (41). Furthermore, SAHA has been reported to suppress cancer cell proliferation through downregulation of cyclin D1 expression in mouse epidermal Cl41 cells (42).

Consistent with the present findings, accumulating evidence indicates that activation of the extrinsic apoptotic pathway represents one of the principal mechanisms by which SAHA induces apoptosis in malignant cells. In agreement with our results, SAHA has been shown to activate this pathway in the acute myeloid leukemia (AML) cell line Kasumi-1 through upregulation of death receptor 4 (DR4) and death receptor 5 (DR5) (43). Similarly, SAHA has been reported to induce apoptosis in 17B and 22B head and neck squamous cell carcinoma (HNSCC) cells by upregulating Fas, DR4, and DR5, followed by activation of caspase-8 and caspase-3 (44). A comparable mechanism has also been described in human hepatocellular carcinoma cell lines (HepG2, Hep3B, and SK-Hep1), further supporting the role of SAHA in triggering death receptor-mediated apoptosis across different malignancies (45). Collectively, these findings support the hypothesis that modulation of death receptor signaling is a conserved mechanism underlying the pro-apoptotic activity of SAHA in diverse cancer cell types. In addition to the extrinsic apoptotic pathway, activation of the intrinsic apoptotic pathway represents another mechanism underlying SAHA-induced apoptosis, as demonstrated in the present study. Consistent with these known proapoptotic effects, SAHA has been reported to increase the Bax/Bcl2 ratio in AtT-20 mouse pituitary tumor cells. Such a mechanism has also been indicated in SAHA-treated A549, Calu-6, HCC-33, and NCI-H69 cells (46, 47). A similar mechanism has also been reported in SAHA-treated human cancer cell lines, including A549, Calu-6, HCC-33, and NCI-H69, in which modulation of Bax and Bcl-2 expression was associated with induction of the intrinsic apoptotic pathway (46). Recent evidence from acute lymphoblastic leukemia models further supports the relevance of apoptosis-related mechanisms as potential therapeutic targets in leukemia (48). Collectively, these observations support the involvement of the mitochondrial apoptotic pathway in the pro-apoptotic activity of SAHA across different tumor types.

In summary, SAHA treatment altered the expression of key regulatory genes involved in cell cycle progression and apoptotic signaling, which was associated with enhanced apoptosis in glioblastoma, medulloblastoma, and acute lymphoblastic leukemia cell lines. As summarized in Table 4 and illustrated in Figures 4-6, the most pronounced gene expression changes were observed in the medulloblastoma cell line, in which genes associated with both the intrinsic and extrinsic apoptotic pathways showed the greatest degree of modulation. Notably, this cell line also exhibited the highest percentage of apoptotic cells in the flow cytometric analysis. The concordance between the gene expression profile and the apoptotic response suggests that coordinated regulation of apoptotic signaling contributes to the enhanced sensitivity of medulloblastoma cells to SAHA. Furthermore, the more pronounced modulation of genes involved in the extrinsic apoptotic pathway may indicate that death receptor-mediated signaling represents a potentially important mechanism underlying the pro-apoptotic effects of SAHA in this experimental model, although additional functional studies are required to confirm this observation.

In particular, the pronounced molecular and apoptotic responses observed in medulloblastoma cells suggest that this tumor type may represent a promising target for future preclinical investigations of SAHA-based therapeutic strategies.

### 5.1. Study Limitations

The present study focused on gene expression analysis by quantitative RT-PCR. Protein expression and functional activation of the investigated signaling pathways were not evaluated. Therefore, complementary analyses such as Western blotting, immunofluorescence, or enzyme activity assays are required to confirm whether the observed transcriptional changes translate into corresponding protein-level alterations.

### 5.2. Conclusions

Our findings demonstrate that SAHA modulates the expression of genes involved in cell cycle regulation as well as the extrinsic and intrinsic apoptotic pathways, leading to induction of apoptosis in glioblastoma, medulloblastoma, and acute lymphoblastic leukemia cell lines. These findings suggest that SAHA exerts antitumor activity through coordinated regulation of multiple molecular pathways and support its potential as a therapeutic candidate for these malignancies. Future investigations, including in vivo models and clinical studies, are needed to confirm these results and evaluate their potential clinical significance.

## Data Availability

The dataset presented in the study is available on request from the corresponding author during submission or after publication.
